# Designing electrochemical microfluidic multiplexed biosensors for on-site applications

**DOI:** 10.1007/s00216-022-04210-4

**Published:** 2022-07-06

**Authors:** Regina T. Glatz, H. Ceren Ates, Hasti Mohsenin, Wilfried Weber, Can Dincer

**Affiliations:** 1 FIT Freiburg Center for Interactive Materials and Bioinspired Technologies, University of Freiburg, 79110 Freiburg, Germany; 2 Department of Microsystems Engineering (IMTEK), Laboratory for Sensors, University of Freiburg, 79110 Freiburg, Germany; 3 Faculty of Biology and Signalling Research Centers BIOSS and CIBSS, University of Freiburg, 79104 Freiburg, Germany

**Keywords:** Multiplexing, Amperometric biosensors, Point-of-care testing, Microfluidics

## Abstract

Clinical assessment based on a single biomarker is in many circumstances not sufficient for adequate diagnosis of a disease or for monitoring its therapy. Multiplexing, the measurement of multiple analytes from one sample and/or of the same target from different samples simultaneously, could enhance the accuracy of the diagnosis of diseases and their therapy success. Thus, there is a great and urgent demand for multiplexed biosensors allowing a low-cost, easy-to-use, and rapid on-site testing. In this work, we present a simple, flexible, and highly scalable strategy for implementing microfluidic multiplexed electrochemical biosensors (BiosensorX). Our technology is able to detect 4, 6, or 8 (different) analytes or samples simultaneously using a sequential design concept: multiple immobilization areas, where the assay components are adsorbed, followed by their individual electrochemical cells, where the amperometric signal readout takes place, within a single microfluidic channel. Here, first we compare vertical and horizontal designs of BiosensorX chips using a model assay. Owing to its easier handling and superior fluidic behavior, the vertical format is chosen as the final multiplexed chip design. Consequently, the feasibility of the BiosensorX for multiplexed on-site testing is successfully demonstrated by measuring meropenem antibiotics via an antibody-free β-lactam assay. The multiplexed biosensor platform introduced can be further extended for the simultaneous detection of other anti-infective agents and/or biomarkers (such as renal or inflammation biomarkers) as well as different (invasive and non-invasive) sample types, which would be a major step towards sepsis management and beyond.

**Graphical Abstract:**

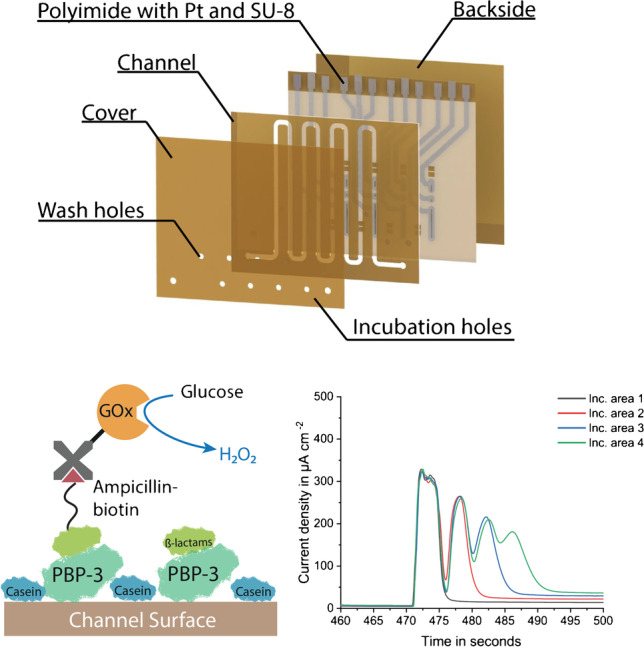

**Supplementary Information:**

The online version contains supplementary material available at 10.1007/s00216-022-04210-4.

## Introduction

For effective and successful treatment of diseases, their early detection and accurate diagnosis are crucial. Biomarkers play an important role in this process since they can indicate the presence, absence, or the severity of various diseases [1–3]. In many circumstances, however, clinical evaluation based on a single biomarker is not enough for adequate diagnostics and therapy monitoring. Multiplexing enables the simultaneous monitoring of multiple analytes and/or samples, which dramatically improves the possibility for diagnosis of many diseases, since detecting multiple biomarkers and their diagnostic correspondence in different biofluids [4] can give us more information about the disease, its status, and treatment [5, 6]. Moreover, patients often suffer from several diseases simultaneously (for example, diabetes and gout) and thus, the simultaneous measurement of several biomarkers (glucose and uric acid) from a single drop of body fluid would dramatically reduce the pain and effort of the patients during self-management of their chronic diseases [7, 8]. The importance of multiplexing has recently increased significantly, as in addition to above-mentioned advantages, it reduces the healthcare costs and enables faster results [9, 10].

Multiplexing is mostly realized by (i) spatial separation of detection areas, (ii) regional separation by discrete regions, for example, of a channel network, or by (iii) the use of different biorecognition or signal-generating elements. In the design of multiplexing with spatial separation of detection areas, the most commonly used format is the separation of the working electrodes (WEs) of an electrochemical cell [5]. With this approach, different molecules can be measured at different WEs, either all with own counter (CE) and reference electrodes (RE) [7, 11] or with shared CEs and REs [12, 13]. This can be further extended by the separation into multiple channels [7, 11], chambers [14], or wells [15]. Although these possibilities are mostly applied for electrochemical detection, using optical detection via labeling with differently colored dyes is an additional option for multiplexing [6, 16]. For the realization of multiplexed biosensors, it is also possible to employ different biorecognition elements, including (monoclonal) antibodies, antigens, enzymes (such as glucose or lactate oxidase), or proteins [5, 7, 13, 14, 16].

There is no single ultimate substrate material for the fabrication of multiplexed sensors, whereas mostly polymers, including PDMS (polydimethylsiloxane) [17], PET (polyethylene terephthalate) [7], polyesters [11], polyimides [13], or nylon membranes [5] are used. In addition, paper-based sensors play a major role in multiplexed biosensors. There, electrodes are often being screen printed using pastes on paper substrates [18, 19], but also stencil printing, inkjet printing, and spray coating are used methods [19]. In addition, there are biosensing platforms that employ beads of different materials as the substrates, on which the reaction takes place. Moreover, glass [20] or CMOS (complementary metal–oxide–semiconductor) [12] substrates are used as the base materials for (multiplexed) biosensing platforms. Dry-film photoresists (DFRs) are very advantageous for building 3D microfluidic structures for biosensors since they are flexible, easy to handle, cheap, and suitable for batch production (also known from flexible electronics). Depending on the application (wearable or on-site) and the sample to be analyzed, additional material characteristics like biocompatibility, bendability, disposability, and compatibility for employed signal transduction method should as well be considered. DFR-based production, in this regard, could be a better alternative for multiplexed sensing.

For multiplexed sensing, electrochemical transduction is one of the most preferred signal readout strategies [21, 22]. Here, either the reaction products can be directly sensed as current or potential in an amperometric or potentiometric method [12] but also different readout techniques like cyclic voltammetry [12], electrochemiluminescence [18], electrochemical impedance spectroscopy [5, 14], or surface-enhanced Raman spectroscopy [23] are employed. Another possibility for multiplexed readout is the optical detection using flourescent dyes [6, 16, 20] or using localized surface plasma resonance (LSPR)-based nanoplasmonic biosensors [24]. Nanoplasmonic sensors can offer high sensitivity, high throughput, label-free, and multiplexed analysis; however, they may suffer from requirement of complex instrumentation and low specificity while working with complex samples. Miniaturization by integrating all components into a one small device [25] and eliminating complex sample preparation without compromising specificity and selectivity [26] would be the two main challenge for optical sensors to be used in the point-of-care. Nevertheless, electrochemical biosensors play a major role in biomolecule detection, not only due to their high accuracy, specificity, sensitivity, and great potential for real sample analysis [27] but also their fast, simple, and low-cost fashion in connection to compact handheld or even wearable analyzers [28].

To fully reveal the potential of multiplexed biosensors in point-of-care applications, we choose therapeutic drug monitoring (TDM) as our proof-of-concept. TDM is the clinical practice of measuring drug levels in blood or other body fluids to understand the drug-response relationship, which allows the individualization of patient’s drug treatment. Individualized dosing of medicines can improve patient outcomes and reduce healthcare costs [29, 30]. In current TDM practice, time-consuming, expensive, and complex chromatographic methods are being used. TDM can be simplified and speeded up by employing biosensors, which are easy to use and provide results in a short time [30–32]. Multiplexed biosensors combine the advantages of multiplexing and biosensing technologies, and are, therefore, optimal candidates for TDM. Multianalyte analysis enables the simultaneous monitoring of several biomarkers or drugs, while multisample analysis helps to generate a cross-correlation database for “well established” blood (plasma) and “less-known” non-invasive samples [4, 30].

In this work, the implementation and in-depth comparison of newly designed electrochemical multiplexed chip designs have been demonstrated for the first time. We designed and developed a single-use, DFR-based microfluidic multiplexed electrochemical biosensor (BiosensorX) platform for various on-site applications. We first explored the basics of design and performance of multiplexed microfluidics. Herein, we tested different formats with a model assay to investigate the possible influences of channel length, pressure drop, and channel alignment on the analytical performance. Consequently, simultaneous quantification of various meropenem concentrations on the same chip was successfully demonstrated as a proof-of-concept study for on-site TDM applications. Last, calibration curves for meropenem antibiotic were obtained with single and multiplexed biosensors to examine the possible cross-contamination between the different incubation areas of the designed multiplexed biosensors.

## Methods

### Design and fabrication of BiosensorX

The detailed explanation of the production of the disposable DFR-based microfluidic biosensors can be found in the Supplementary Information. In short, a polyimide substrate is patterned with a metallization (i.e., platinum) using a lift-off process, followed by defining the functional surface with the photoresist SU-8 [33, 34]. The chips are then finalized by lamination of several previously developed DFR layers in order to create the microfluidic channel.

**Fig. 1:**
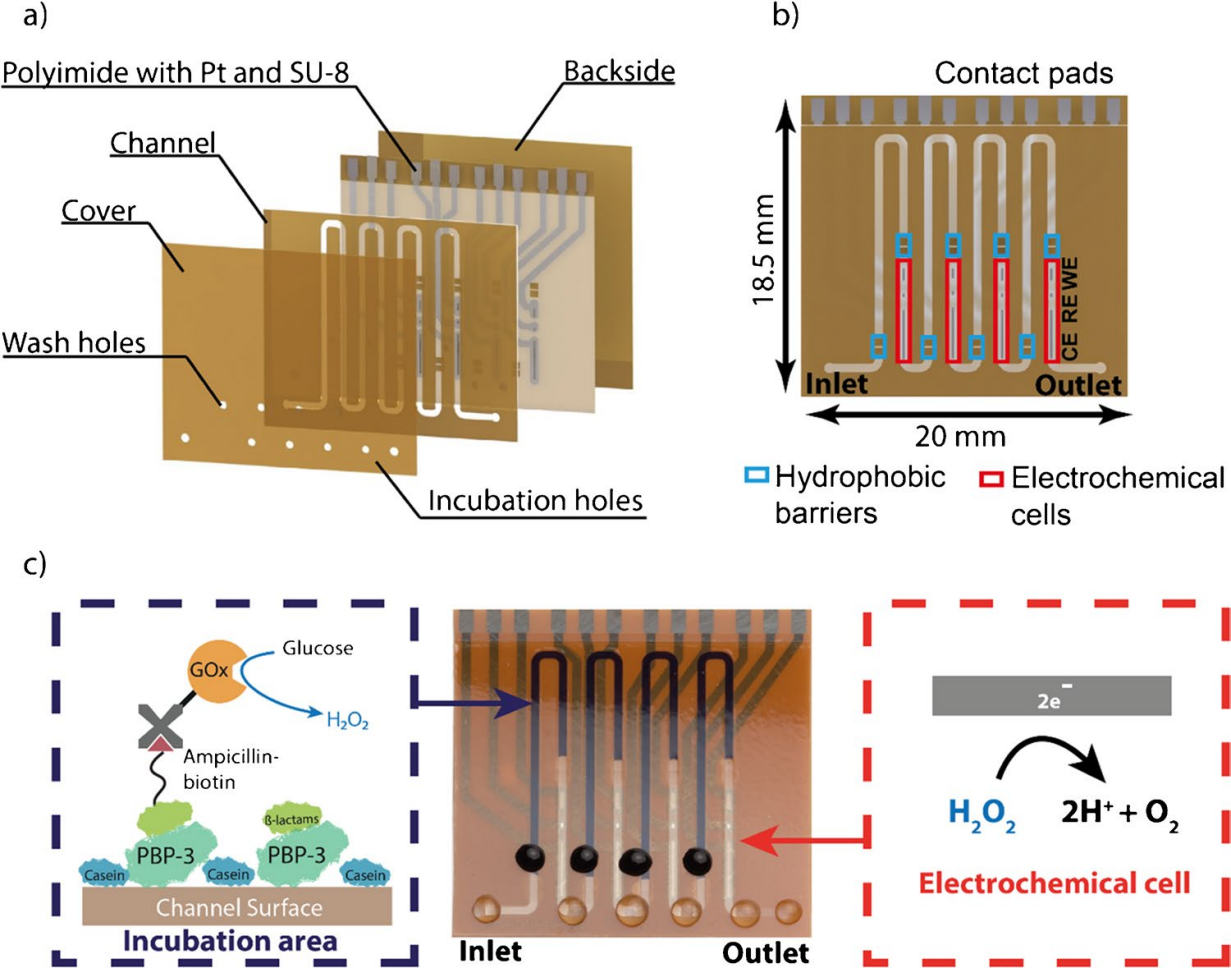
Rendered chip images using SolidWorks (Dassault Systems SolidWorks Corp., France), showing **a** different layers before lamination of 4-plex chip design and **b** the final chip with the description of its main components. **c** Picture of 4-plex vertical chip showing assay being proceeded in incubation area and the electrochemical reaction happening at the working electrode. The incubation holes of the chips are the ones covered with blue colored liquid, and the washing holes and inlet and outlet are covered by transparent PBS droplets

The channel configuration is the same for both single analyte/sample (miLab) and multiplexed (Biosensor X) biosensors (Fig. 1b): An incubation area, where the biomolecules are immobilized and an electrochemical cell, where the amperometric readout takes place. The electrochemical cell consists of a RE, WE, where the reaction takes place, and CE, which electrically stabilizes the cell. The incubation area and the electrochemical cell are separated by a hydrophobic stopping barrier, filled with Teflon. This barrier prevents the biomolecules from entering and contaminating the electrochemical cell. In the multiplexed designs, two barriers are placed after each other to improve the stopping behavior compared to the former design.

In the multiplexed designs, several of these units (incubation area and electrochemical cell) are placed sequentially one after the other in a single channel. The designs presented here include 4, 6, and 8 units. Each incubation area is equipped with its own incubation hole (colored droplet in Fig. 1c), at the beginning of the incubation area to introduce the biofluids properly into the channel, and a washing hole (transparent droplet in Fig. 1c) to enable proper washing of these individual areas. In addition to individual inlet and outlets, each biosensor chip also contains a common inlet and outlet, which allows homogeneous pumping of the measurement solutions through each immobilization area. For the electrical connection to the measurement setup, both single analyte/sample and multiplexed biosensors (miLab and BiosensorX) are equipped with contact pads accordingly (Fig. 1b).

The multiplexed designs can be made in horizontal (Fig. S2a,b) and vertical (Fig. 1a, b) channel orientation. The design benefit of the vertical versions is the shorter total channel length, which enables lower total pressure drop compared to the horizontal versions (Table 1). The biosensors can be further distinguished by their readout strategy: In the “MUX” designs (Fig. S2c,d), where a potentiostat with multiplexer is used for the measurement, all channels have one common RE and CE, and individual WEs. With this configuration, it is possible to measure 4, 6, and 8 analytes/samples simultaneously. Only in the 4-plex designs (Fig. 1a, b, Fig. S2c,d), all channels have individual RE, CE, and WEs and can be read out via a multichannel (i.e., 4-channel) potentiostat which is also used for measuring miLab chips.

**Table 1:** Comparison of different multiplexed sensor designs (Hor: horizontal, Vert: vertical) and miLab

BiosensorX	4-plex Hor	4-plex Vert	6-plex Hor	6-plex Vert	8-plex Hor	8-plex Vert	miLab
Length (mm)	18.5	18.5	18.5	22	22	22	10
# of Incubation areas	4	4	6	6	8	8	1
Chips per wafer	30	30	30	26	26	26	130
Total channel length (mm)	172	124	238	227	310	274	25
Total channel volume (μl)	5.5	4	7.6	7.2	9.9	8.8	0.8
Immobilization region length (mm)	21	19	21	20	21	20	16.4
Immobilization region volume (μl)	0.67	0.61	0.67	0.64	0.67	0.64	0.52
Pressure drop in channel (kPa)	2.86	2.06	3.95	3.77	5.15	4.55	0.41

### Measurement procedure

Before starting the measurement, the electrodes are preconditioned in 10 mM PBS (pH 7.4) to ensure same measurement conditions in all WEs for sensitive and reproducible results and to electrochemically remove possible residues from the electrodes. The measurement itself is also started in 10 mM PBS until the signal saturates, then it is changed to a 40-mM glucose solution (40 mM in 10 mM PBS (Sigma Aldrich, USA), substrate for the glucose oxidase (GOx) enzyme used). 2 - and 5-min stop-flow protocols are applied to achieve a signal amplification (Fig. S5). During the stop phase, hydrogen peroxide (H_2_O_2_) produced by the enzymatic reaction is accumulated in the channel, which is then flushed over the electrochemical cell when restarting the flow, to generate the typical rectangular-like current peaks for stop-flow measurements [35]. In the case of multiplexed biosensors, the primary peaks correspond to the accumulation of electrochemical active species in the immobilization area during the stop phase. During the “flow” phase, these species are also passing through neighboring electrochemical cells, in addition to their own individual electrochemical cell, which results in the following current peaks.

### System characterization

#### Comparison of different designs with model assay

After fabricating different versions of multiplexed biosensors, their fluidic and electrochemical performance is studied using a model assay with streptavidin glucose-oxidase (StrGO_*x*_). Herein, 1 μl of 10 μg ml^−1^ StrGO_*x*_ [32] is pipetted to each of the incubation holes of the multiplexed chips for 1-h incubation. Subsequently, the washing step using 50 μl of 0.05% TWEEN® 20 in 10 mM PBS (Sigma Aldrich, USA) for each incubation area is performed. After the washing step, StrGO_*x*_-functionalized chips are placed into the custom-made chip holder for the amperometric measurement (see “Measurement procedure”).

### Proof-of-concept for TDM of antibiotics

#### Antibody-free meropenem assay incubation

The on-chip assay procedure starts with the adsorption of 250 μg ml^−1^ penicillin binding protein 3 (PBP-3, produced and purified in-house) on the channel surface for 1 h [32]. This step is followed by a 20-min incubation of biotin-free casein (85R-108, Fitzgerald, USA) for blocking the remaining active surface sites. The third step of the assay consists of the competitive binding of different concentrations of the target β-lactam antibiotic (Meropenem, Fresenius Kabi Deutschland, Bad Homburg) in the sample solution and 0.2 μg ml^−1^ biotinylated ampicillin (synthesized in-house) as competitor to the PBP-3 in a 1-h incubation. Finally, 10 μg ml^−1^ StrGO_*x*_ (FGI 65R S125, Biozol, Germany) is incubated for 15 min in order to generate the electrochemical signal. After each incubation step, all incubation areas are washed separately. For the on-chip meropenem calibration, known concentrations of meropenem antibiotic (10^−4^, 10^−3^, 10^−2^, 0.1, 1, 10, and 100 μg ml^−1^) are spiked into 10 mM PBS. Calibration measurements are then executed by incubating the four channels of the sensor with four subsequent concentrations of meropenem. A calibration curve with the same concentrations is also drawn with the single analyte/sample biosensor, to investigate the possible cross-contamination between the individual incubation areas of the multiplexed biosensors designed.

## Results and discussion

### Comparison of different designs using the model assay

**Fig. 2:**
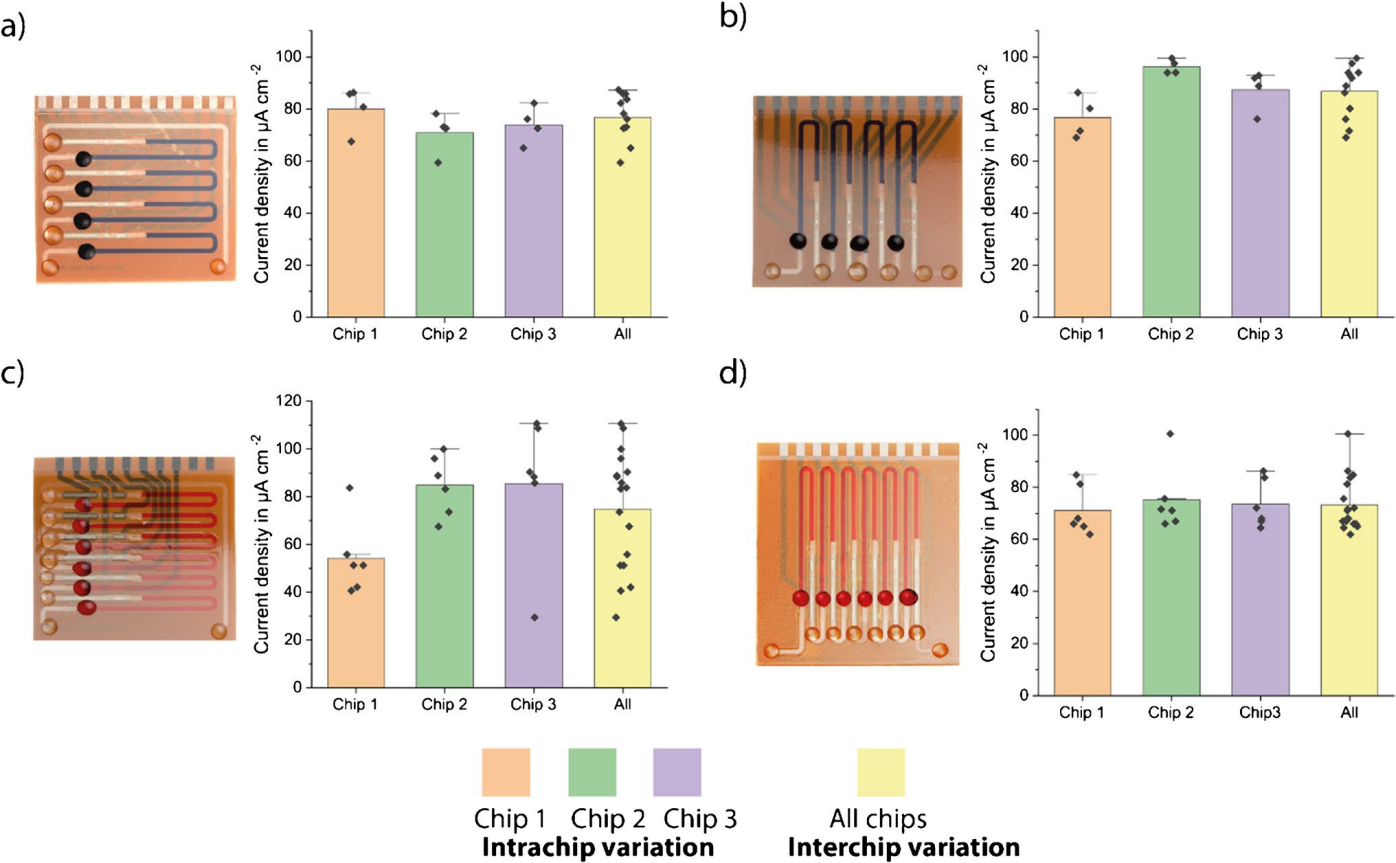
Pictures and characterization results using the model assay with 10 μg ml^−*1*^ StrGO_*x*_ of **a** 4-plex horizontal, **b** 4-plex vertical, **c** 6-plex horizontal, and **d** 6-plex vertical designs of BiosensorX. Bar plots of *n* = 3 replicates. The error bars represent ± standard deviation (SD)

During the initial characterization, horizontal versions showed higher inter- and intra-chip deviations than the vertical versions (Fig. 2). We speculated that these higher deviations are mainly due to two reasons: inadequate washing procedure and bubble formation inside the channels during the measurement. Problems were encountered notably with the horizontal versions during washing. When the last (top) incubation zone was washed, the end of the channel also filled with wash buffer; that is why uniform washing could not be guaranteed, since the equal amount of wash buffer flowing through each incubation area was not ensured at all times. In the vertical versions, however, it could be shown that all channels were washed equally. Secondly, in the horizontal design, especially the 6- and 8-analyte versions, bubbles were formed during the measurement, most probably due to high pressure drop in the channel. The presence of bubbles falsifies the results of the stop-flow measurements since the fluid flow does not stop immediately with the start of the stop phase.

With the 4- and 6-plex designs, three replicates were performed each measurement. During the measurements with the 8-plex design, on the other hand, some electrical problems due to the potentiostat used were encountered, which leads to only two valid measurements for horizontal version (Fig. S8). The results were investigated concerning intra- and inter-chips variations. Intra-chip variation means the deviations between the individual readout sections of one chip, whereas inter-chip variation means the deviation between different chips. These values are important to understand the sources of deviations, and to investigate whether the approach of multiple incubation areas in one single channel is feasible for multiplexed analysis.

Overall, the vertical versions were found to be easier to handle during the washing and measurement processes than the horizontal versions. From these findings, we put our focus on to the vertical versions and performed further characterizations by using the vertical designs.

**Fig. 3:**
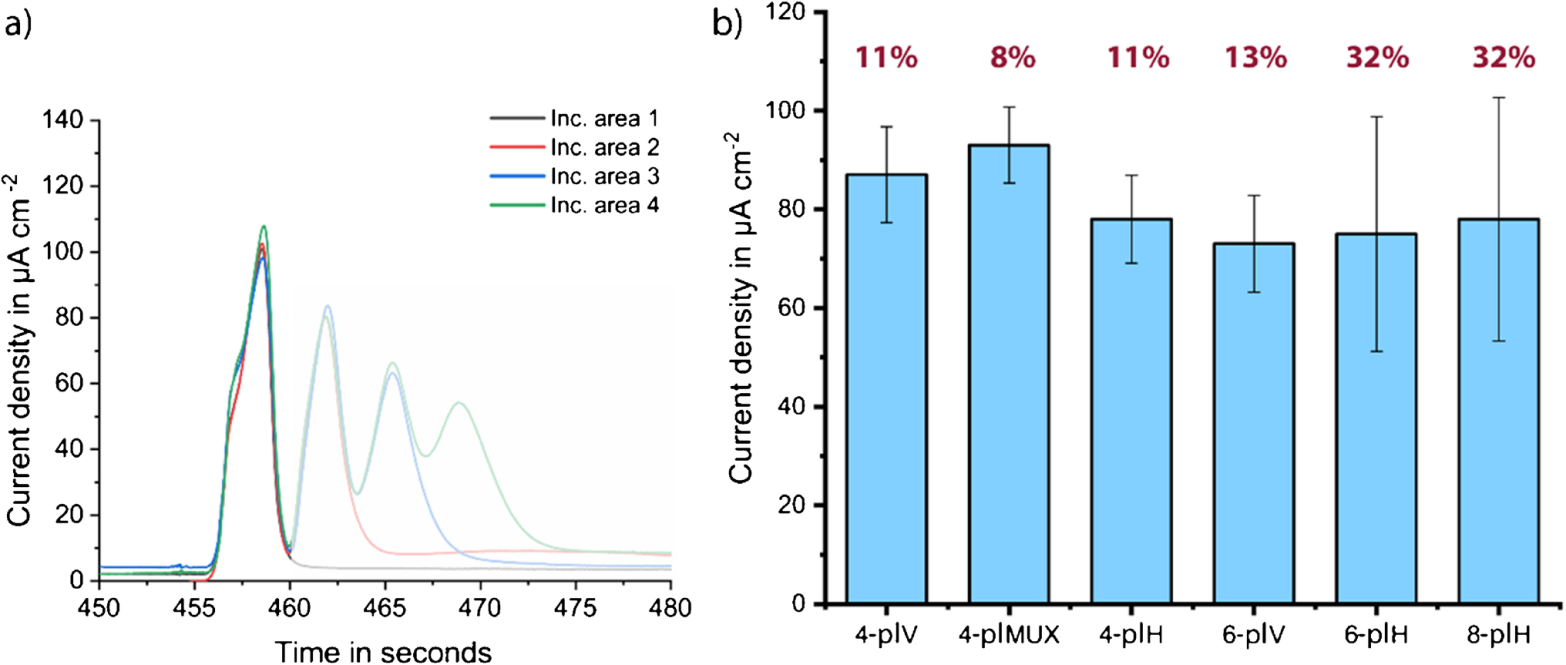
Comparison of all tested chip designs using the model assay with 10 μg ml^−*1*^ StrGO_*x*_. **a** An example measurement (4-plex vertical) to show non-saturated current peaks (more measurement peaks in Fig. S6) and **b** bar plots of *n* = 3 (*n* = 2 for 8-plex) replicates, and the error bars represent ± standard deviation (SD), including coefficients of variation (CVs) in percentage. Vertical versions show lower CVs and thus, better fluidic behavior

The shape of current signals obtained during the initial characterization hints a possibility of a nonuniform biomolecule coating (Fig. S6). Consequently, the comparison tests were repeated with a higher concentration of StrGO_*x*_ (next section) to ensure that the deviations occurred solely due to the design differences and not due to limited biomolecule concentration. Designs having the same number of incubation areas were compared (Fig. 3).

### Final characterization of vertical designs

**Fig. 4:**
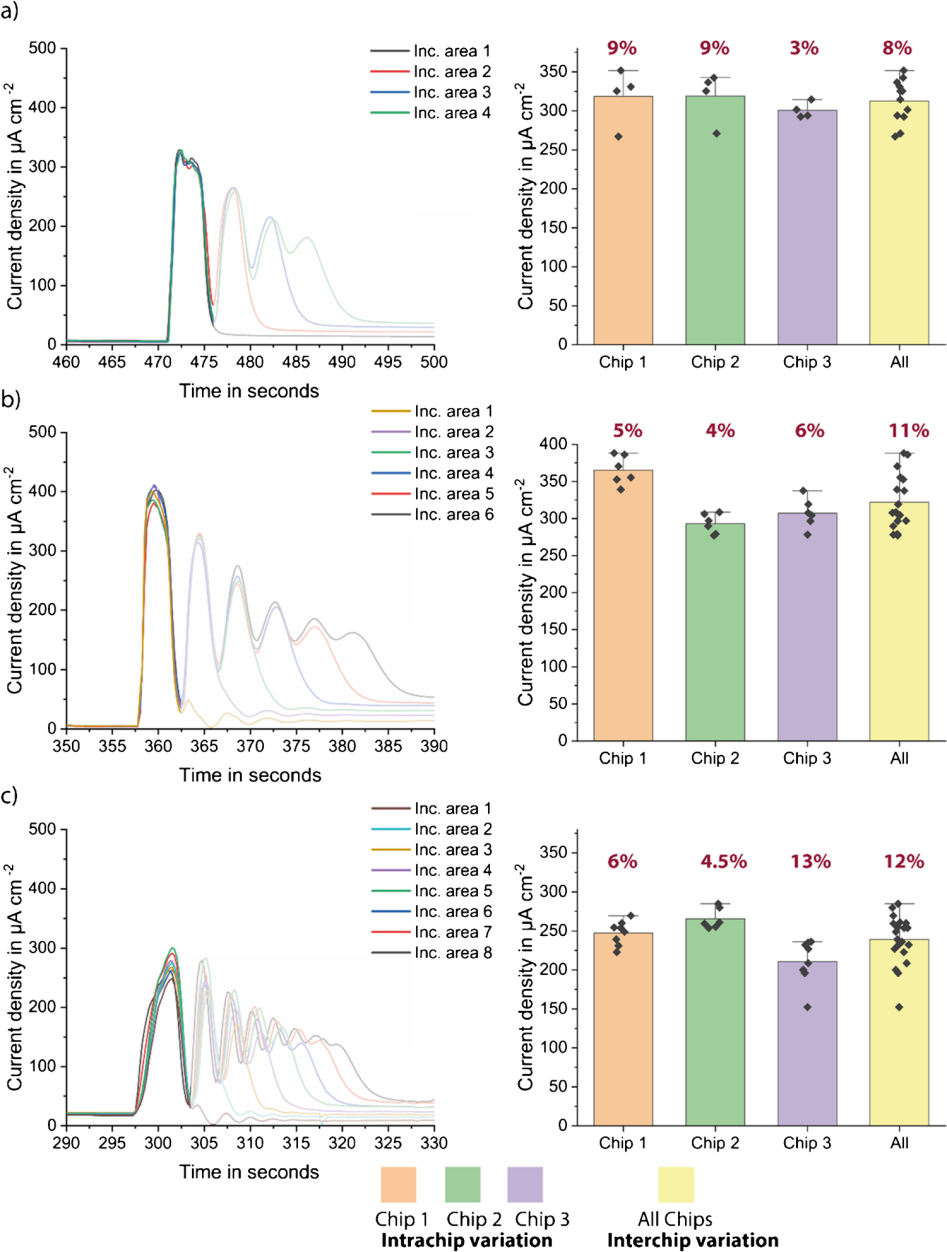
Model assay results of vertical designs, peak shapes (left), and measured current densities after 2-min stop-flow protocol, with inter- and intra-chip variations (right) for **a** 4-plex vertical, **b** 6-plex vertical, and **c** 8-plex vertical. Bar plot of *n* = 3 replicates. Error bars represent the outlier range and standard deviation written as percentage

Herein, the model assay tests were repeated with the higher concentration of StrGO_*x*_ using previously selected vertical designs. Chips were incubated with 200 μg ml^−1^ StrGO_*x*_ for 1 h and measured with 2-min stop-flow protocol. The results of the vertical designs showed that the 4- and 6-plex biosensors behave very similarly, showing similar signal heights and standard deviations, intra-chip, and inter-chip (Fig. 4a, b). On the 8-plex version, however, the peaks were lower and wider, suggesting some fluidic problems such as bubbles and partially controlled flow rate inside the channel (Fig. 4c). These problems could be related to the high back pressure inside the channel (Table 1); therefore, the 8-plex version still needs further optimization. Moreover, 4-plex versions have the advantage of smaller size, which results in more chips per wafer and therefore lower cost per chip (Table S1). The 4- and 6-plex versions also have shorter total channel lengths, resulting in lower total pressure drop which enables better fluidic behavior, such as less bubble formation and uniform fluid flow. After final comparison of the three vertical designs, 4-plex vertical versions were selected for the following proof-of-principle measurements.

### Proof-of-principle by calibration of meropenem assay using BiosensorX

To demonstrate the multiplexed measurement capability of the newest design of our BiosensorX, we compared the calibration curves of meropenem in physiological PBS, obtained by our single and multiplexed biosensor. The results were fitted with a 4-parameter logistic fit [36, 37], resulting limit-of-detection (LOD) values of 22.5 ng ml^−1^ for miLab and 28.6 ng ml^−1^ for BiosensorX.

**Fig. 5:**
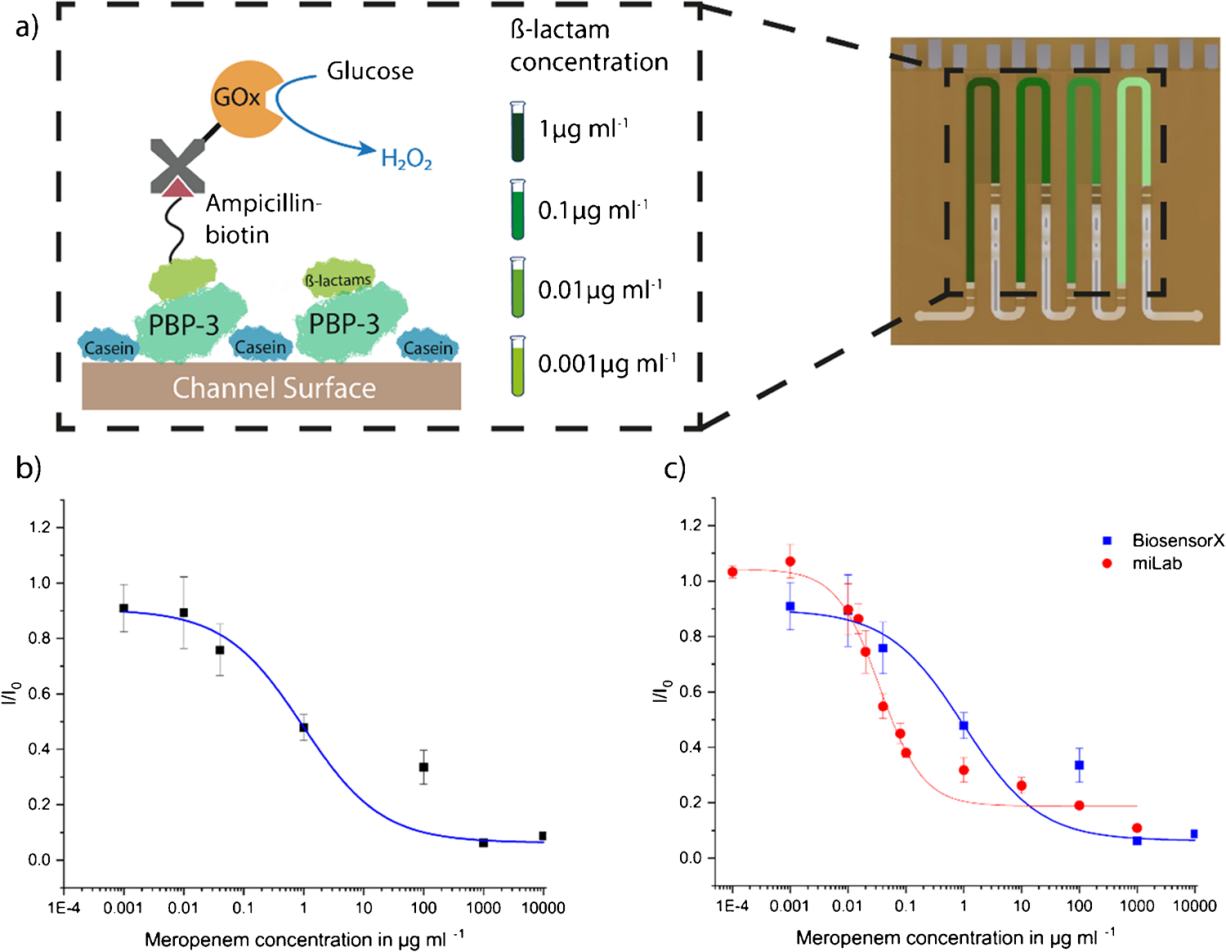
On-chip meropenem calibration in 10 mM PBS using miLab and BiosensorX chips. **a** Antibody-free assay protocol consisting of four steps: 1. PBP-3-binding to surface, 2. casein blocking, 3. competitive binding of meropenem and biotinylated ampicillin, and 4. incubation of signal-generating molecule streptavidin-glucose oxidase. The on-chip assay calibration was performed using 4-plex BiosensorX where different concentrations of meropenem were incubated with the competitor into each immobilization area. Obtained calibration curves of BiosensorX (**b**) and BiosensorX together with miLab (**c**). Both calibration curves were analyzed using 4-parameter logistic fit, from which LODs of 22.5 ng ml^−1^ for miLab and 28.6 ng ml^−1^ for BiosensorX were calculated

Given the almost identical LODs obtained with both miLab and BiosensorX chips, we concluded that it is possible to simultaneously gauge different concentrations on the same chip, without having cross-contamination between consecutive immobilization areas. Although we obtained almost identical LODs, we observed a one-order of magnitude shift in the working range of the assay (Fig. 5c). We speculated that the shift in the operational window can be caused by different length of incubation areas and different flow speed during measurements. For the miLab chip, the flow speed during measurements was 20 μl min^−1^, for BiosensorX; however, 10 μl min^−1^ was used.

## Conclusion and outlook

In this work, we successfully designed and fabricated different microfluidic multiplexed electrochemical biosensors (up to 8 analytes/samples), based on dry-film photoresists, in vertical and horizontal designs for on-site testing. DFRs have the advantage of being easy to produce, suitable for batch production, low cost, and flexible and are, therefore, an optimal candidate for creating microfluidic biosensors. Combining multiplexed sample analysis with electrochemical readout makes the sensors highly specific and sensitive with high accuracy and low detection limits and great potential for multiplexed point-of-care testing.

In most electrochemical biosensors, the biomolecules for signal generation are directly immobilized to the electrode surface, which easily can lead to electrode fouling, if no protective coating is applied. In our biosensing strategy, however, the immobilization area and electrochemical cell are strictly separated by using two hydrophobic stopping barriers. This on one hand enables automatic metering of the fluid and on the other hand prevents the electrochemical cell from being contaminated and moreover allows for analyzing complex biofluids (whole blood, urine, saliva, etc.) with our biosensor platform, without losing its sensitivity [32].

Changing from horizontal design, being used in the previous study [32], to vertical channel design facilitates better fluidic behavior and easier handling during assay and measurement procedures. By using common counter and reference electrodes (“MUX” readout), the chip size could be reduced, which results in lower fabrication costs and simpler readout and enables multiplexed detection up to 8 samples/analytes. The vertical 4- and 6-plex versions showed very promising CVs lower than 5% for model assay tests. Moreover, a calibration curve of an antibody-free β-lactam assay for detection of meropenem antibiotics was successfully performed in miLab and 4-plex BiosensorX, obtaining very similar LODs of 22.5 ng ml^−1^ and 28.6 ng ml^−1^ for miLab and BiosensorX, respectively.

Extension of single-sample/analyte measurements to multisample/analyte analysis brings some challenges with respect to handling, fluidic characteristics, and signal readout; for which further optimization is necessary. Our future work will deal with the following: (i) An easier handling of the biosensor could be enabled by improving the stopping barriers’ behavior and the washing procedure by increasing size or distance of barriers and design a better washing adapter to wash all channels simultaneously. (ii) The 8-plex BiosensorX showed some fluidic challenges, which need to be solved to be able to run 8-analyte/sample measurements. For example, this could be achieved by changing flow rate, improving fluidic connection, and by different tubing material (such as Teflon) for less bubble formation. (iii) Using the BiosensorX platform in clinical applications, the measurement setup needs to be further miniaturized, automated, and/or easier to use. Herein, a smartphone-compatible small potentiostat for signal readout along with a micropump for the stop-flow protocol could be used [38]. The BiosensorX presented in this work is capable of detecting several analytes simultaneously and can be further extended to detect several biomarkers and anti-infective agents in different sample types on a single chip, which is a huge step towards fast and easy therapeutic drug management at the point care.

## Supplementary Information

ESM 1 (PDF 2.90 MB)
