# Supplementary material for: Designing electrochemical microfluidic multiplexed biosensors for on-site applications
Source: Anal Bioanal Chem. 2022 Jul 6;414(22):6531–40. doi: 10.1007/s00216-022-04210-4 (PMC9411084; doi:10.1007/s00216-022-04210-4)
Supplement: Supplementary file 1 — (PDF 2.90 MB) [file 216_2022_4210_MOESM1_ESM.pdf]

Supporting Information

Designing electrochemical microfluidic multiplexed biosensors for on-site applications

Regina T. Glatz[1, 2, †], H. Ceren Ates[1, 2, †], Hasti Mohsenin[3], Wilfried Weber[3], and Can Dincer[1, 2, *]

[1] *University of Freiburg, FIT Freiburg Center for Interactive Materials and Bioinspired Technologies, 79110 Freiburg, Germany*

[2] *University of Freiburg, Department of Microsystems Engineering (IMTEK), Laboratory for Sensors, 79110 Freiburg, Germany*

[3] *University of Freiburg, Faculty of Biology and Signalling Research Centers BIOSS and CIBSS, 79104 Freiburg, Germany*

Corresponding author

Can Dincer (dincer@imtek.de)

[†]Equally contributed authors

1. Wafer-level fabrication process of biosensors

The fabrication procedure of the miLab and Multilab biosensors was already described in [1] and [2] in detail and obeys a strict protocol consisting of ten steps (**Figure S1**). In the following, these steps will be shortly explained in the order they are executed during fabrication. After each step, the wafers are washed with DI-water and dried with pressurized air.

1.1. Cutting *of polyimide and copper etching*

First, the 6” wafers are cut from the Pyralux AP8545 (DuPont, USA) substrate using a pair of scissors. The polyimide film is covered with copper on both sides which is removed in a bubble etch tank (PA104, MEGA Electronics, UK) filled with 20% sodium persulfate at a temperature of 45 °C for 1 hour.

1.2. Spin coating and development of MA-N 1420

At this step, a special resist allowing a lift-off process is used in order to form the metallization area for creating the contact pads and electrodes. Herein, 2-3 µl of the resist MA-N1420 (Micro resist technology GmbH, Germany) is spin-coated on the wafer and then it is soft baked for 2 minutes at 100 °C. This is followed by a 2-minute UV exposure on an exposure unit (Hellas, Bungard Elektronik, Germany) using a foil mask (for metallization) (MKD Kramer, Germany) and then development in two baths of ma-D 533s (Micro resist technology GmbH, Germany).

1.3. Platinum deposition and lift-off step

To realize the metallization, 200 nm of platinum (Pt) is deposited onto the wafer by a physical vapor deposition (PVD) process in the clean room at IMTEK, University of Freiburg. For removing the excess Pt, the wafers are dipped in a remover bath, containing ma-R 404s (Micro resist technology GmbH, Germany), on a shaker for 40 minutes.

1.4. Formation of insulation layer with SU-8

At this step, the epoxy resist SU-8 (3005, Microchem, USA) is applied on the wafer to define the electrode areas / contact pads, forming the wells for Teflon stopping barrier and isolating the not-active metal parts. First, the wafers are soft-baked in an oven at 120 °C for 10 minutes prior to spin coating 6 ml of the resist onto the wafer. This step is followed by two baking steps on different hotplates, for 2 minutes at 65°C and 3 minutes at 90°C, respectively. After letting the wafers dry overnight in closed carriers, the UV-exposure for 1:20 minutes using a foil mask (for isolation) is performed, followed by development in three baths of 1-methoxy-2-propyl acetate solutions (Merck KGaA, Germany) and one bath of isopropanol is conducted.

After the washing step, the wafers are hard-baked for 3 hours at 160°C starting from / ending at the room temperature in an oven (Binder, Germany).

1.5. Plasma cleaning step

To remove the SU-8 residues, the wafers are placed into a standard plasma unit (Tetra30-LF-PC, Diener, Germany). The Pt electrodes are "cleaned" by using 300 W low-frequency power with 100% oxygen flow, at a flow rate-controlled pressure of 0.4 mbar, for a total plasma reaction time of 3 minutes.

1.6. Silver deposition and chlorination of reference electrodes

To create on-chip Ag/AgCl reference electrodes on wafer level, an electrodeposition step is employed. To protect the contact pads of the chips during this step, they are covered with a UV-sensitive tape. The silver deposition takes place in an alkaline silver cyanide solution (Arguna S, Umicore, Germany), immersed in an ultrasonic bath (Sonorex Super 10 P, Bandelin, Germany). The bulk contact pad of the wafer is the cathode of the process, while as anode a bare silver wire, immersed to the Arguna S solution is used. At the next step, the wafer is placed in an 0.1 % potassium chloride (KCl) solution for partial chlorination of the silver deposited to create on-chip reference electrodes. Finally, the UV-tape is removed after a short UV-illumination.

1.7. Dispensing of Teflon for formation of hydrophobic stopping barriers

To prevent the bioreagents from getting into the area of the electrochemical cell and thus, from poisoning the electrodes, hydrophobic stopping barriers (single for miLab and multiple for BiosensorX) are formed. Therefore, a small drop of a 3% Teflon (AF 1600, DuPont, USA) in a FC-75 solution is dispensed into the wells (formed by the SU-8), using a hand dispenser (1500 XL, Nordson EFD).

1.8. Production of DFR layers

For the realization of the microfluidic channels, the dry-film photoresist (DFR) Pyralux PC1025® (DuPont, USA) is used. The DFR is cut in appropriate pieces, and then exposed in the standard vacuum exposure unit using the respective foil masks. For one wafer, four layers are needed: a channel, a cover and two backside layers. Last two ones prevent the chips from bending during the final backing step.

Upon illumination, the layers are developed in two dishes filled with 1% sodium carbonate (Na_2CO_3), placed in an ultrasonic bath. To stop the development, prevent an over development and release stress from the layers, they are shaked for one minute in a 1% hydrochloric acid (HCl) bath. After that, the layers are carefully but thoroughly rinsed with DI-water and dried pressurized air. Before continuing with

lamination, the layers should be kept for some hours (better for couple days) at room temperature to reduce their stickiness while covered with a lid to prevent any dust formation.

1.9. Lamination of DFR layers

Herein, the wafer is sticked on an overhead foil and the appropriate DFR layers are aligned under a microscope, starting from the channel layer, then the two backsides and finally the cover. The layers are laminated one after the other with a standard hot roll laminator (HRL 350, Ozatec). It is important to remove the shiny foil of each Pyralux PC layer before continuing with the lamination of the next layer.

1.10. Chip cutting and hard bake

After all layers are laminated, the wafer can be finalized by pulling the last two protective foils from top and bottom and cutting the wafer in stripes, using a pair of scissors. Finally, the chips are baked in an oven for 3 hours at 160 °C starting from / ending at the room temperature. After that step, the chips are ready to be used.

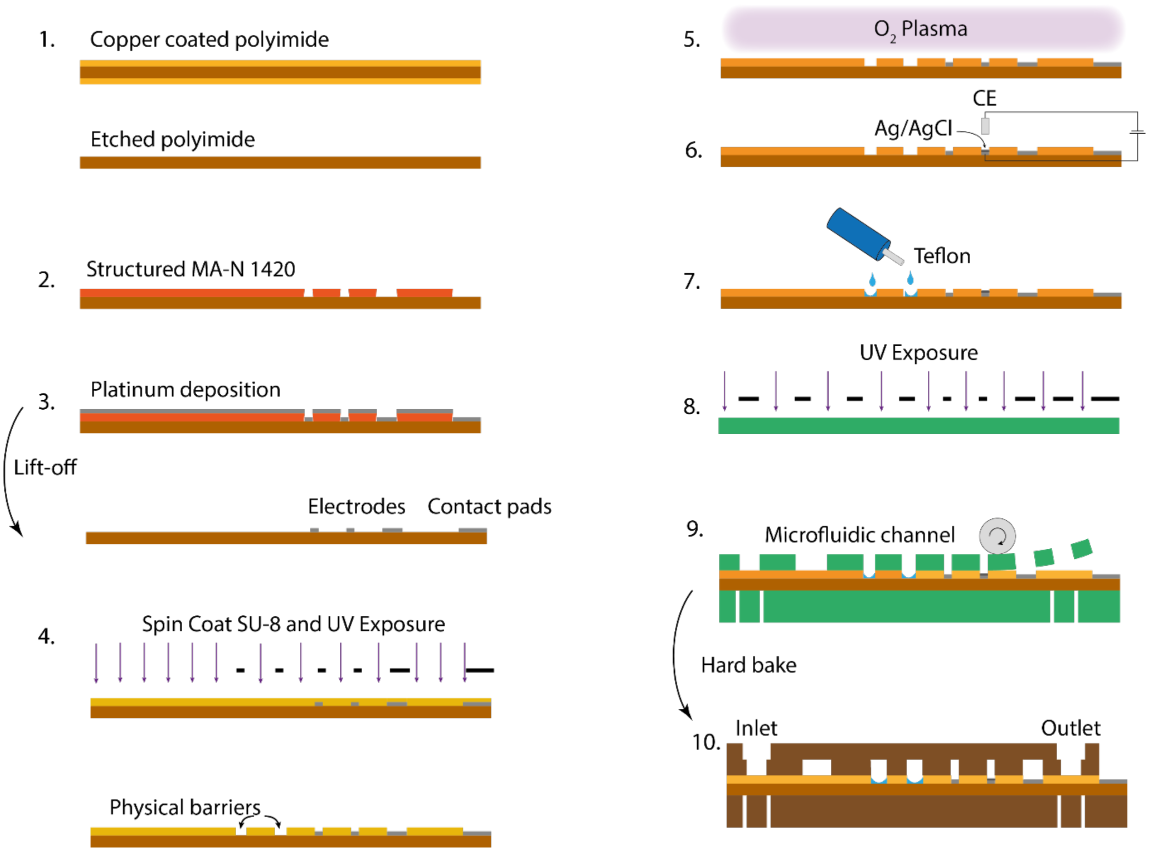

Figure S1. *Schematic representation of fabrication process divided in 10 steps: 1. Etching of copper coated polyimide. 2. Spin-coating and structuring of MA-N photoresist, 3. Platinum deposition for the formation of*

metallization, 4. Spin-coating and development of SU-8 as insulation layer, 5. O_2 plasma step to clean SU-8 residues, 6. Wafer-level Ag/AgCl deposition for creating reference electrodes, 7. Forming stopping barriers by dispensing Teflon, 8. Exposure and development of DFR layers, 9. Lamination of DFR layers, 10. Hard bake for 3 hours, adapted from [1].

2. Biosensor designs

Additionally to the 4-plex vertical Biosensor, discussed in the main text, several chip designs have been estimated and measured, namely 4-plex horizontal (**Figure S2a and b**), 6-plex vertical (**Figure S2c and d)** and horizontal, 8-plex vertical and horizontal and milab chip (**Figure S2e and f**).

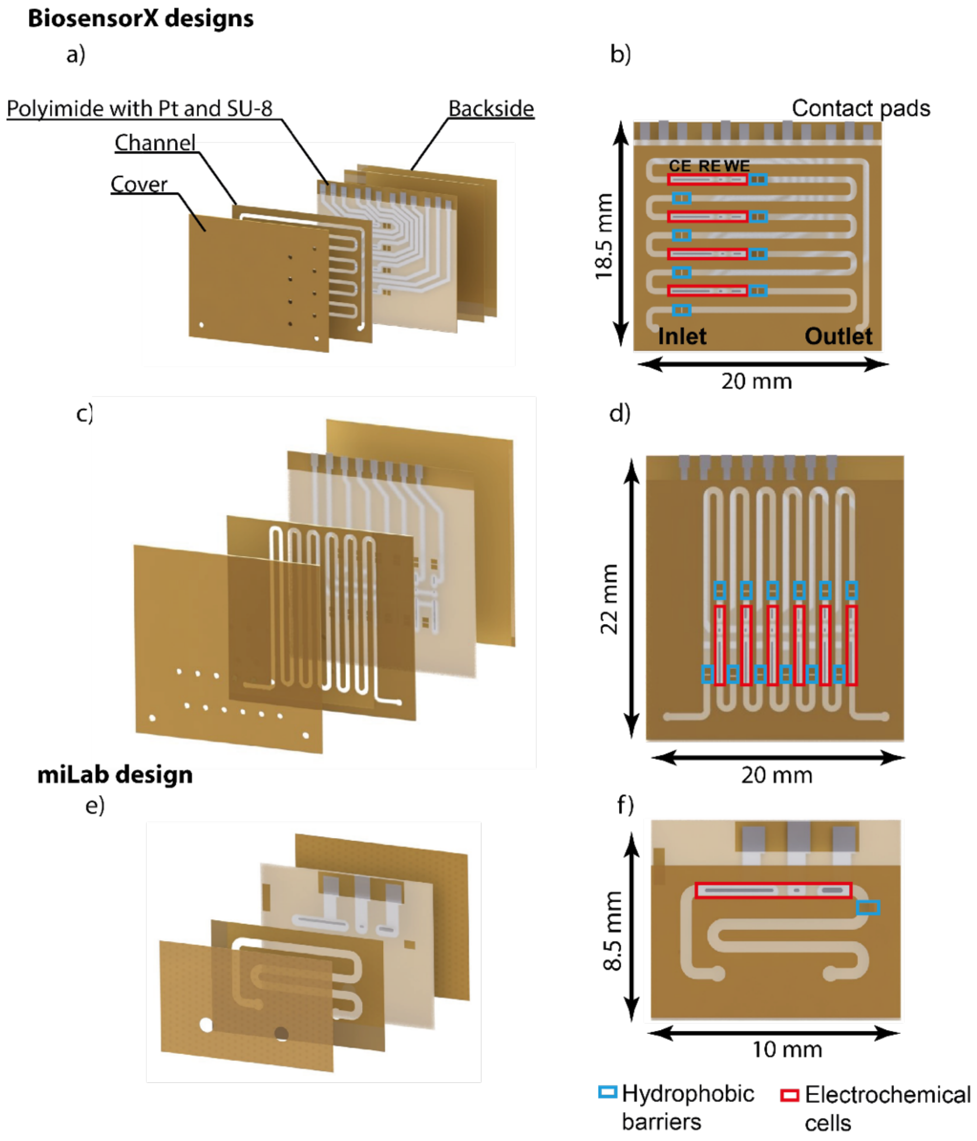

Figure S2. *Additional Biosensor designs to 4-plex vertical presented in the main text, each showing rendered layers and assembled chips, respectively.* ***a)*** *and* ***b)*** *4-plex horizontal,* ***c)*** *and* ***d)*** *6-plex vertical and* ***e)*** *and* ***f)*** *miLab chip.*

3. Cost estimation of biosensors designed and fabricated in this study

To estimate the fabrication costs, a batch of four wafers produced under research laboratory conditions is considered. **Table S1** shows the cost calculation including all fabrication steps, and total costs per measurement channel for all biosensor designs: miLab chip (two biosensors per chip) and the different designs of BiosensorX (18.5 mm and 22 mm). The costs per incubation area range from 3.400 € (8-plex version) to 2.948 € (4-plex version).

Table S1*. Cost estimation for the fabrication of different chip designs.*

Fabrication step	Material	Costs per chip				
		miLab	4-plex Hor/Vert	6-plex Hor	6-plex Vert	8-plex Hor/Vert
**Substrate**	Pyralux AP8545	0.194 €	0.420 €	0.420 €	0.486 €	0.488 €
**Lift-off resist**	ma-N 1420	0.048 €	0.104 €	0.102 €	0.120 €	0.120 €
**Metallization**	Pt PVD	0.960 €	2.080 €	2.082 €	2.400 €	2.400 €
**Isolation**	SU-8 3005	0.134 €	0.292 €	0.288 €	0.336 €	0.336 €
**Reference electrodes**	Ag and KCl	<0.001 €				
**Stopping barrier**	3% Teflon	<0.001 €				
**Dry-film photoresist**	Pyralux PC1025	0.020 €	0.044 €	0.044€	0.048 €	0.005 €
**Total:**		**1.360 €**	**2.948 €**	**2.948 €**	**3.402 €**	**3.400 €**

4. Chip connection and integration

For the assay incubation and electrochemical signal readout, different components are necessary to ensure an easy handling and accurate results. These components, therefore, must perfectly fit together and be suitable for the designed multiplexed biosensors.

During the assay incubation, a washing step is needed to remove unbound biomolecules from the channel after each consecutive biomolecule incubation. Herein, a vacuum adapter (**Figure S3**) is used for removing the excess/unbound biomolecules from the channel inlet via vacuum applied. These custom-made adapters are designed corresponding to the chip layout and produced by 3D printing (Ultimaker 3 Extended, Ultimaker, Netherlands) using acrylonitrile butadiene styrene (ABS), which is durable and hard polymer. The connection between the biosensor and the vacuum adapter is ensured with stainless steel pipes (Nordson EFD, Germany) and vacuum cups (Nordson EFD, Germany) added at the top of the pipes for a tight sealing.

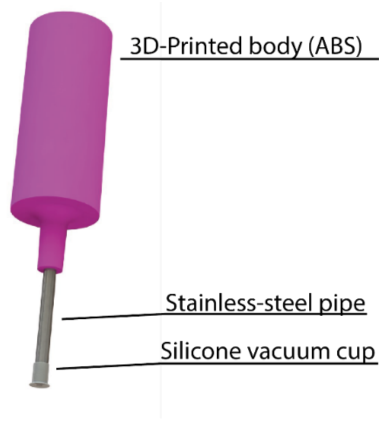

Figure S3. *Vacuum adapter, used for washing step for BiosensorX. Top part* ***(left)*** *is connected to vacuum, while the vacuum cup* ***(right)*** *is adjusted to the washing inlet on the biosensor. The adapter was produced by 3D printing (Ultimaker 3 Extended, Ultimaker, Netherlands) using acrylonitrile butadiene styrene (ABS), which is a durable and hard polymer. The legs are stainless steel pipes (Nordson EFD, Germany) and for a tight sealing to the chip vacuum cups (Nordson EFD, Germany) are added at the top of the pipes.*

For the electrical and fluidic connection of the chips to the measurement setup, a lasered PMMA sheet (***Figure S4*****a**) (Präzisions-Acrylglas transparent, Architekturbedarf.de, Germany) is glued on the chip, which seals the incubation and wash holes and additionally stabilizes the chip against bending. The chip is then placed to the custom-made chip holder (**Figure S4c**) and fluidically connected with the fluidic adapter (***Figure S4*****d**). These two components are milled out of a PMMA plate and finalized with magnets

(Supermagnete, Webcraft GmbH, Germany), stainless steel pipes and silicone tubes (Deutsch & Neumann, Germany). For the electrical connection a printed circuit board (PCB, BetaLayout, Germany) (**Figure S4c**) is needed, which is placed in the chip holder. PCBs used in this work are designed using the EAGLE PCB Design Software (CadSoft Computer GmbH, Germany). It is equipped with a ground socket (Hirschmann Test & Measurement, Germany) and a 37-pin D-sub connector (Assmann WSW Components Inc., USA), which enables an easy plug-in connection to the potentiostat. In addition, there is a rotary backlock connector on the PCBs to easily connect the chips electrically. The measurement setup is completed by either a multichannel potentiostat (MultiEmStat3, PalmSens BV, The Netherlands), or (depending on the chip design) a potentiostat with an 8-channel multiplexer (EmStatMUX8, PalmSens BV, The Netherlands), a syringe pump (PHD ULTRA 4400, Harvard Apparatus, USA) and a notebook with the measurement software (**Figure S5**).

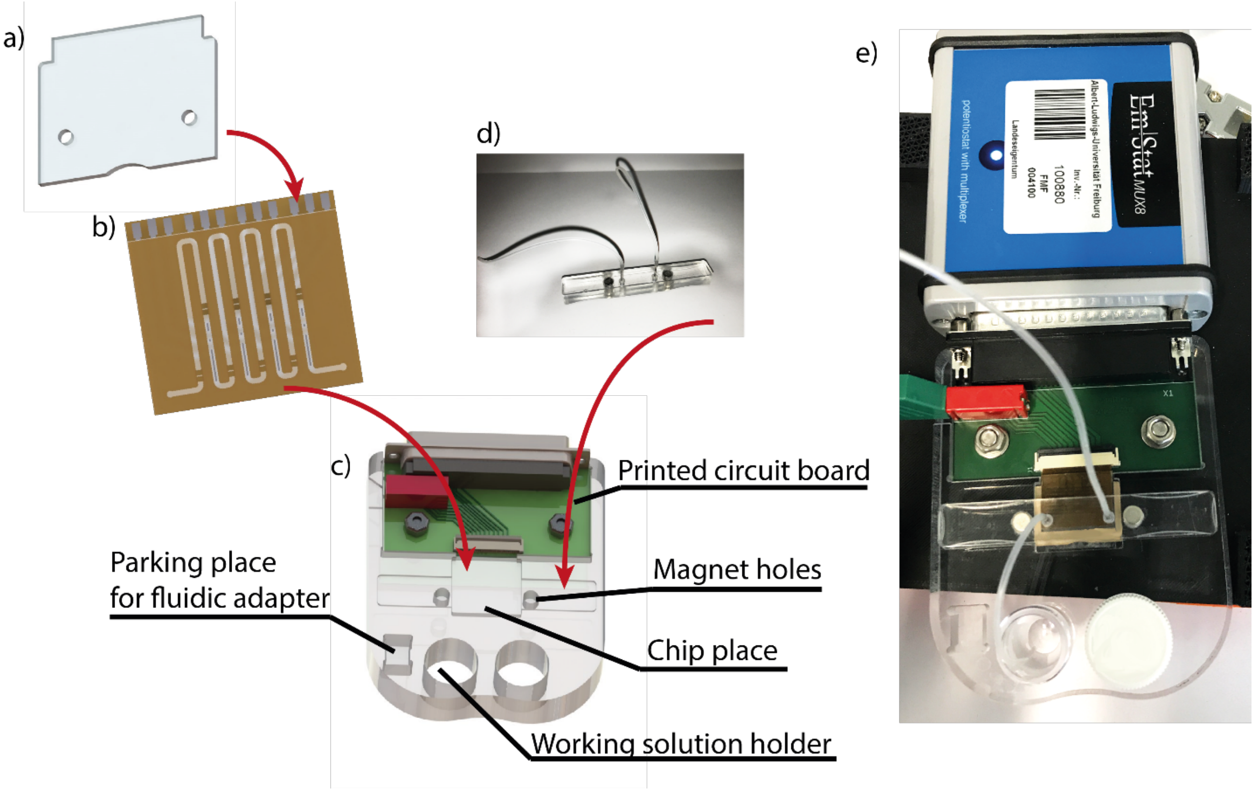

Figure S4. *System components, including **a)** PMMA sheet, lasered out of PMMA plate, to seal all inlets/outlets except the common inlet/outlet for the measurement, **b)** BiosensorX chip to be measured, **c)** chip holder with PCB, **d)** custom-made fluidic adapter and **e)** all components assembled and connected to potentiostat. Herein, the holder and the fluidic adapter were both designed in SolidWorks 2017 (Dassault Systemes SolidWorks Corp., France) and milled out of PMMA (Acrylglas GS, Architekturbedarf.de, Germany).*

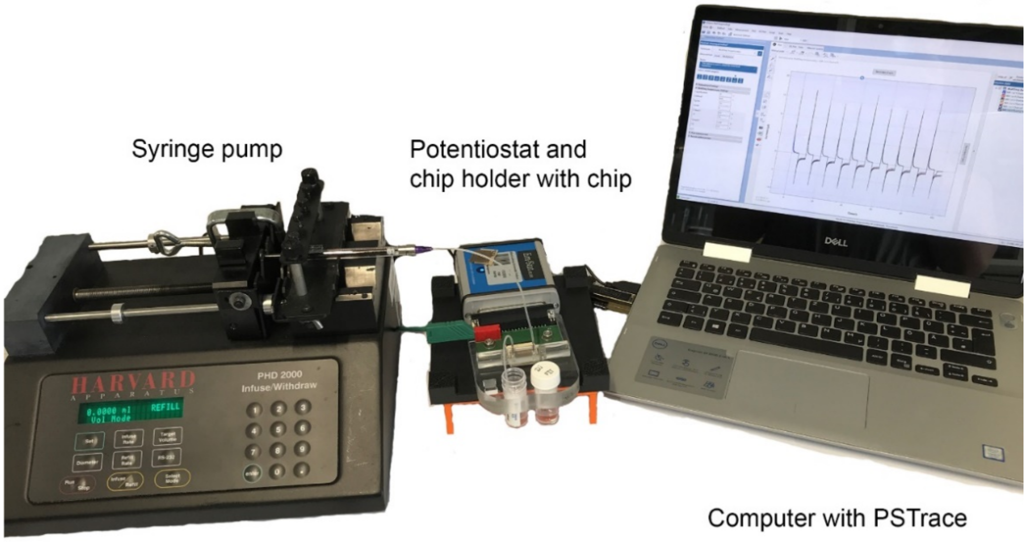

Figure S5*. Whole measurement setup, consisting of syringe pump, custom-made holder with PCB, connected to potentiostat, working solutions, and a notebook with measurement software, connected via USB to the potentiostat.*

A syringe pump (PHD ULTRA 4400, Harvard Apparatus, USA), potentiostat (MultiEmStat3, PalmSens BV, The Netherlands) and notebook with the measurement software (MultiTrace, PalmSens BV, The Netherlands) complete the measurement setup (**Figure S5**). During the measurement procedure, a stop-flow protocol is applied to the biosensors in order to obtain a signal amplification (**Figure S6**).

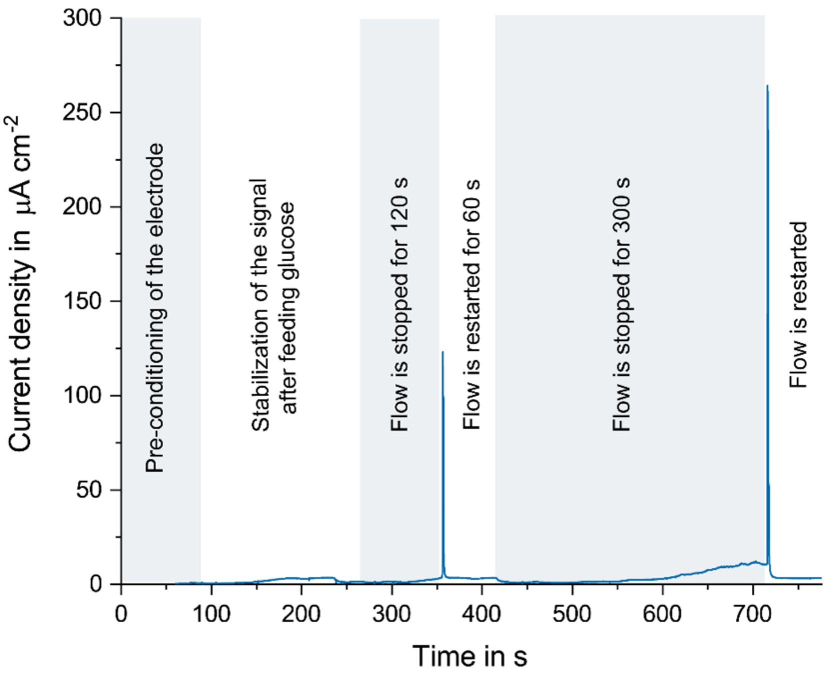

Figure S6. *Stop-flow protocol applied to the microfluidic biosensors for the electrochemical measurement and signal amplification.*

5. Initial results using multiplexed biosensors

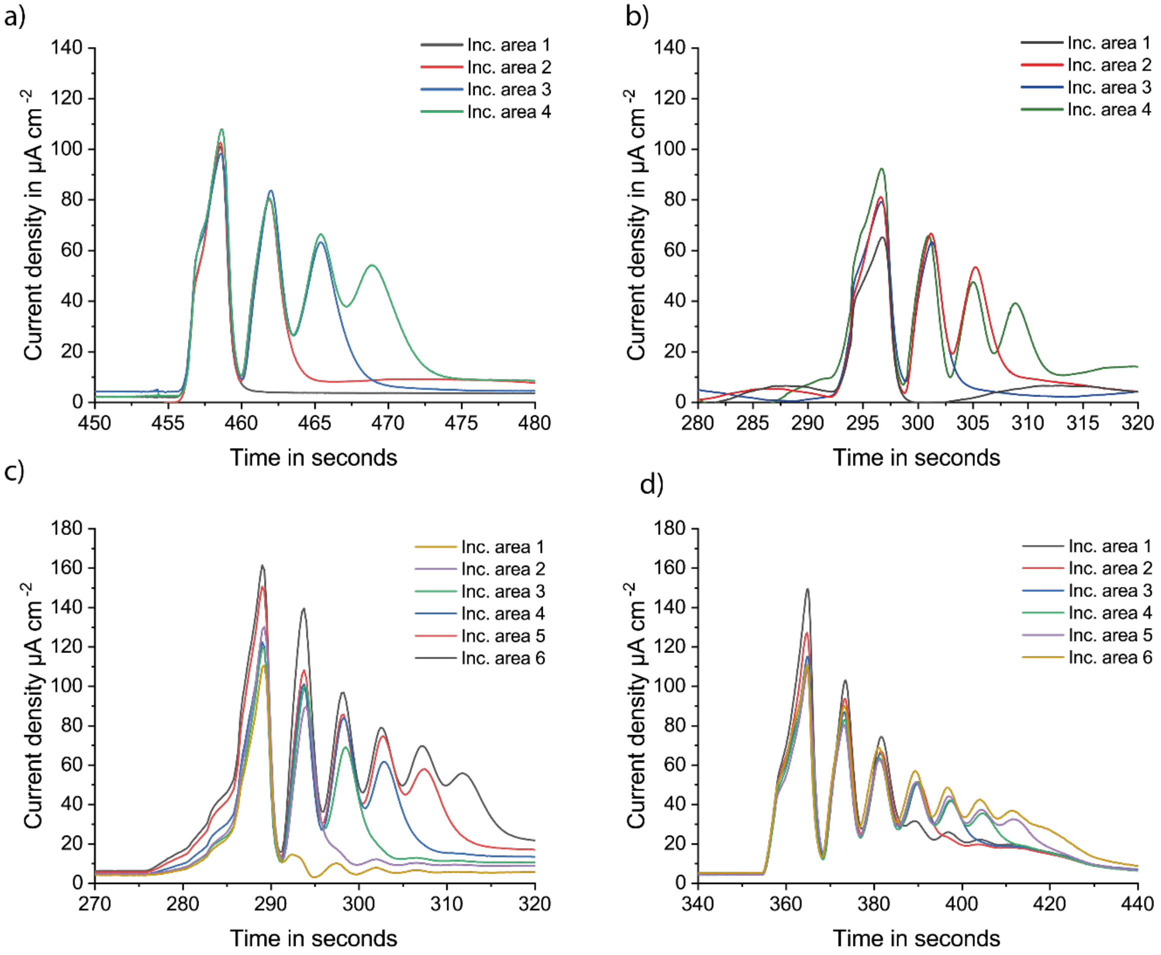

Figure S7. *Initial characterization plots obtained with different multiplexed chip designs:* ***a)*** *4-plex vertical,* ***b)*** *4-plex horizontal,* ***c)*** *6-plex vertical and* ***d)*** *6-plex horizontal. Obtained current peaks did not show a rectangular shaped signal. This indicates that the channels were not completely saturated with the signaling biomolecule employed: StrGOx. Therefore, we repeated these tests (****Figure 4****) with a higher concentration of StrGOx (200 µg ml^{-1}), to ensure the deviations were solely due to the design differences and not due to limited biomolecule concentration.*

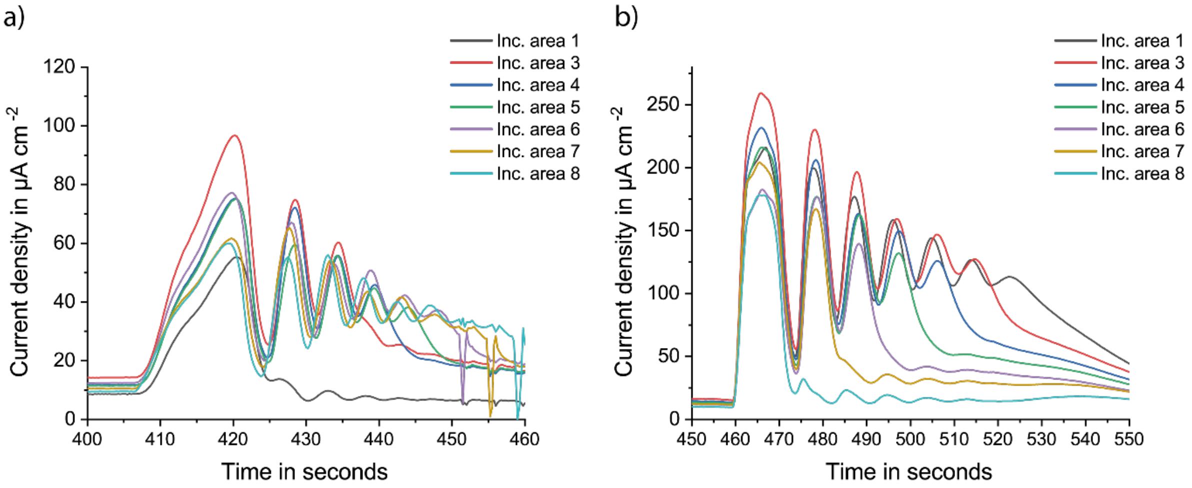

Figure S8. *Initial characterization plots obtained with 8-plex BiosensorX.* ***a)*** *8-plex horizontal incubated with 10 µg ml^{-1} StrGOx and* ***b)*** *8-plex vertical incubated with 200 µg ml^{-1} StrGOx. There are no results for incubation area 2, due to electrical problems with potentiostat.*

6. Limit of detection

The limit of detection (LOD) of an assay describes the lowest measurable analyte concentration and is also referred to as "sensitivity". It is defined through a certainty level of 3.3 σ (corresponds to 95%), where σ is the standard deviation of the blank value. For most assays, the correlation between the analyte concentration and the signal measured is non-linear and can be fitted with a 4- or 5- parameter logistic fit. Here, 4-parametric logistic model was employed to determine the LOD by using the limit of blank (LOB) (**equation 1**) which is the highest apparent analyte concentration expected, when blank samples are measured:

$$LOB_{signal} = \ mean_{blank} + 1.645\ SD_{blank} \quad \text{(Eq. 1)}$$

and

$$y = LOD_{signal} = LOB_{signal} + 1.654\ SD_{low\ concentration\ sample}. \quad \text{(Eq. 2)}$$

To calculate the LOD, the equation of the 4-parametric logistic fit is then used; **equation 3**:

$$y = A_{min} + \frac{A_{max}-A_{min}}{1+(\frac{x}{x_0})^p} \quad \text{(Eq. 3)}$$

which can be solved for x by inserting LOD_{signal}; **equation 4** [3]:

$$LOD_{concentration} = x_0 \cdot (LOD_{Signal} - A_{max})^{\frac{1}{p}} \quad \text{(Eq. 4)}$$

where x_0 is the inflection point of the curve, A_{max} the response at low concentration and p the slope factor of the fitting curve [4,5].

References

1. Bruch R. Multiplexed miRNA Biosensor for Pediatric Cancer Diagnostics.

2. Bruch R, Kling A, Urban GA, Dincer C. Dry film photoresist-based electrochemical microfluidic biosensor platform: Device fabrication, on-chip assay preparation, and system operation. Journal of Visualized Experiments. 2017;2017:1–7.

3. Findlay JWA, Dillard RF. Appropriate calibration curve fitting in ligand binding assays. AAPS Journal. 2007;9:E260–7.

4. Armbruster DA, Pry T. Limit of blank, limit of detection and limit of quantitation. Clin Biochem Rev. 2008;29 Suppl 1:S49-52.

5. Long GL, Winefordner JD. Report. 1983;55.
